# Comparison of the efficacy of human umbilical cord mesenchymal stem cells conditioned medium and platelet-rich plasma on the hippocampus of STZ–induced rat model of Alzheimer’s disease: A behavioral and stereological study

**DOI:** 10.1016/j.ibneur.2023.09.006

**Published:** 2023-09-14

**Authors:** Amin Firoozi, Aliakbar Alizadeh, Asadollah Zarifkar, Tahereh Esmaeilpour, Mohammad Reza Namavar, Omid Alavi, Farzaneh Dehghani

**Affiliations:** a Department of Anatomy, School of Medicine, Shiraz University of Medical Sciences, Shiraz, Iran; b Histomorphometry & Stereology Research Center, Shiraz University of Medical Sciences, Shiraz, Iran; c Department of Tissue Engineering and Applied Cell Sciences, School of Advanced Medical Sciences and Technologies, Shiraz University of Medical Sciences, Shiraz, Iran; d Neuroscience Research Center and Department of Physiology, Shiraz University of Medical Sciences, Shiraz, Iran; e Clinical Neurology Research Center, Shiraz University of Medical Sciences, Shiraz, Iran

**Keywords:** Alzheimer’s disease, Platelet-rich plasma, Human umbilical cord mesenchymal stem cells, Stereology, Hippocampus

## Abstract

**Introduction:**

Alzheimer’s disease (AD) is accompanied by progressive cognitive disorders and memory loss. This study aims to determine the combined effects of conditioned medium of human umbilical cord mesenchymal stem cells (CM) and platelet-rich plasma (PRP) on AD model rats.

**Methods:**

Forty-eight male Sprague Dawley rats were classified into 6 groups: Control, Sham, AD, and three treatment groups. AD was induced by streptozotocin(STZ; 3 mg/kg, intracerebroventricular (ICV)) and the treatment groups received injections of CM [(200 µl, intraperitoneally (i.p.), and/or PRP (100 µl, intravenously(i.v)] for 8 days. Behavioral tests (Morris water maze and novel objective recognition) were used to assess learning ability and memory. At the end of the behavioral tests, the rats were sacrificed and their brain was entirely removed, sectioned, and stained with cresyl violet. The hippocampus volume and number of neurons were evaluated by stereological techniques.

**Results:**

In the AD group, the discrimination ratio, time spent in the target zone, volume of Cornu Ammonis1 (CA1) and Dentate Gyrus (DG), and the number of pyramidal and granular cells decreased significantly compared to the Sham group. The mentioned parameters increased in the CM and CM+PRP groups compared to the AD group (p < 0.01). PRP did not have any noticeable effect on the examined parameters.

**Conclusions:**

CM may be beneficial in the treatment of AD as it led to better improvement in STZ-induced learning and memory impairments as well as the structure of the hippocampus.

## 1 Introduction

Alzheimer’s disease (AD) is a neurodegenerative disease associated with neuronal cell loss, progressive cognitive disorders, loss of memory, and behavioral changes (Doan et al., 2017, Tromp et al., 2015). The prevalence of this disease rises with age after the age of 65 (Trevisan et al., 2019), making it a common disorder among elderly people (van der Lee et al., 2018). Molecular and epidemiological studies have suggested the role of gene mutations, abnormal proteins, and environmental and endogenous factors in the increasing prevalence of AD (Nacmias et al., 2018, Killin et al., 2016). Excessive amounts of beta-amyloid (Aβ) (Storey and Cappai, 1999, Nunan and Small, 2000) and hyperphosphorylated tau proteins (also called Neurofibrillary tangles (NFTs)) are the primary biomarker of AD which are strongly correlated with the degree of dementia in AD (Brion, 1998). Changes in the neocortex by deposition of Aβ can induce degeneration of neurons through the corticohippocampal connection in AD (Baek et al., 2022).

Some studies have shown a 25% reduction in hippocampal volume and the number of neurons in AD patients compared to the control subjects (Vijayakumar and Vijayakumar, 2013, Andrade-Moraes et al., 2013). Dendritic spines of hippocampal pyramidal cells play an important role in memory storage; which decrease in AD (Mijalkov et al., 2021). The decline in the number of pyramidal cells in the hippocampus is the basic neuropathology of AD (Mann, 1996). Previous studies have shown higher vulnerability of Cornu Ammonis1 (CA1) in AD as its cell population exhibits higher reduction than other parts of the hippocampus (Padurariu et al., 2012, Tang et al., 2009). The dentate gyrus (DG) is a specific part of the hippocampus which receives input from the neocortex through the perforant pathway (Kandel et al., 2000). The pyramidal and granular cells are the most important neurons in the hippocampus with a prominent role in memory and learning (Pléau et al., 2021, Huerta et al., 2000).

Despite the huge efforts based on drugs and non-drug options to develop an efficient treatment for AD, no definitive therapy has been approved for this disorder. Some articles have suggested the key role of growth factors in neuron plasticity and recovery against neuronal degeneration (Sims et al., 2022). Moreover, the level of growth factors decreases in AD (Luppi et al., 2009). Therefore, it could be helpful to develop a novel treatment option involving several growth factors. In this regard, some growth factor-producing treatments such as conditioned medium (CM) and platelet-rich plasma (PRP) could be helpful.

Numerous reports have shown that CM contains antioxidants, growth factors, cytokines, and chemokines which can promote cell migration, angiogenesis, accelerate wound healing, and prevent cell aging (Sriramulu et al., 2018, Arutyunyan et al., 2016, Zhang et al., 2017). Studies have revealed the presence of transforming growth factor-β (TGF-β), insulin-like growth factor1 (IGF-1), vascular endothelial growth factor (VEGF), and brain-derived neurotrophic factor (BDNF) in the umbilical cord stem cell culture media (Salgado et al., 2010). These factors are responsible for the neurogenic functions and neuronal support to improve neuronal functions and reverse the synaptic plasticity in neurons of the hippocampus (Ruiz de Almodovar et al., 2009).

Another treatment for AD in this study is PRP which contains large numbers of platelets over its basal amount in the blood (Ferrari et al., 1987). It can promote tissue regeneration due to the presence of intracellular growth factors like platelet-derived growth factors (PDGF) and TGF-β, by releasing them over a long period (Marx et al., 1998, Sánchez et al., 2003).

The aim of the current study was the assessment of potential effects of CM and/or PRP on the memory and cognition of AD model rats. For this reason, we used the stereological technique to obtain 3D information from 2D histological sections which is more reliable than other quantitative methods such as morphometry.

## 2 Material and methods

### 2.1 Animals & experimental design

Forty-eight adult Sprague Dawley male rats (230–250 g) were supplied from the Comparative and Experimental Medical Center of Shiraz University of Medical Sciences and kept for about two weeks to adapt to the conditions of the research center. Rats were housed in clean solitary cages with free access to water and food. The temperature was kept at 22 °C in a 12/12 light/dark cycle. The experiments were carried out according to the ethical considerations of the NIH guide and ethical principles of working with animals regulated by Shiraz University of Medical Sciences (SUMS). The study was also approved by the SUMS Ethics Committee (Ethic code: *IR.SUMS.REC 1400.047.*). Rats were randomly separated into six groups:

1. Control group: Including healthy rats with no intervention.
2. Sham group: Animals in this group underwent the same surgery process as other groups without Alzheimer’s induction; they received 5 µl buffer citrate microinjection into each lateral ventricle.
3. AD group: Alzheimer’s disease was induced by slow streptozotocin (STZ) injection (Sigma-Aldrich, St. Louis, MO, USA) (3 mg/kg dissolved in 5 µl of buffer citrate) using a Hamilton syringe into each lateral ventricle according to the stereotaxis technique on the first and third day. After the AD induction, the rats were acclimated in individual cages for one week.
4. CM group: After AD induction, animals received 200 µl of CM per day for 8 days intraperitoneally (i.p) (Mehrabadi et al., 2020).
5. PRP group: After AD induction, animals received 100 µl of PRP per day for 8 days intravenously (i.v) through the tail vein (Moghadam et al., 2017).
6. CM + PRP group: After AD induction, followed by treatment with simultaneous injection and dosage of CM and PRP.

**Fig. 1:**
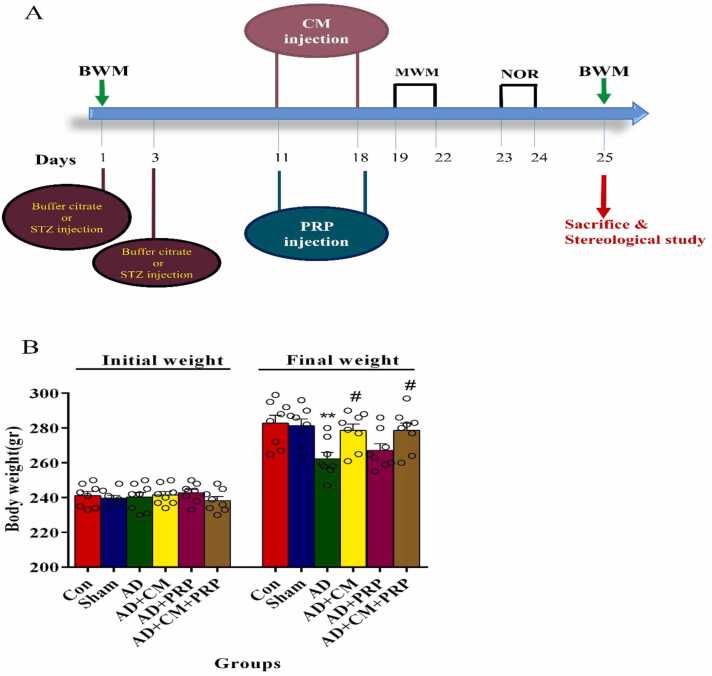
(A) Schematic representation of the experimental design. Rats received STZ or buffer citrate (in the sham group) on the first and third days of the experiment. After one week of the last injection of STZ, the treatment groups received CM and/or PRP for eight days. The day after behavioral tests, the animals were sacrificed for stereological study. (B) The body weight of animals is shown as mean ± SEM (n = 8). * * Significant difference with the Sham group (p < 0.001). # Significant difference with AD group (p < 0.01). STZ: Streptozotocin, Con: Control, AD: Alzheimer’s Disease, CM: Conditioned Medium, PRP: Platelet-Rich Plasma, MWM: Morris Water Maze, NOR: Novel Objective Recognition, BWM: Body Weight Measurement.

After the behavioral tests, on day 25, animals were sacrificed, perfused and brain samples were extracted for stereological study (Fig. 1A).

### 2.2 Surgical procedure

After anesthetizing rats with ketamine (75 mg/kg) and xylazine (10 mg/kg)/i.p., the sham and AD model animals were restrained onto a stereotaxic apparatus and received a bilateral injection of 5 µl STZ or buffer citrate (in the sham group) in ventricles using a Hamilton microsyringe (Bonaduz, Switzerland). The rats were placed in the following direction, relative to the bregma: ± 1.5 mm medial-lateral, + 0.8 mm anterior-posteriorly, and 3.5 mm dorsoventral. Then, they were sacrificed after behavioral tests and their brains were entirely removed by perfusion.

### 2.3 CM preparation

The human umbilical cord was isolated under sterile conditions (Provided by Zeinab Hospital, Gynecology, and Obstetrics Hospital in Shiraz, which is approved by the SUMS Ethics Committee (Ethic code: *IR.SUMS.REC 1400.047.*)) and cleaned with phosphate-buffered saline (PBS) (Gibco, Germany) under a sterile laminar hood. The cord was then cut into smaller pieces (3–5 mm) and arteries and veins were removed by longitudinal sections to make sure all the mesenchymal cells are from Wharton Jelly. The segments were incubated in flasks containing 15% fetal bovine serum (FBS) (Gibco, Germany), Dulbecco's Modified Eagle Medium (DMEM) (Asagen, Iran), and 1% penicillin/streptomycin (P/S) at 5% carbon dioxide and 37 °C. The flask was left for a week, thus, the cells had enough time to attach and grow at the bottom of the flask. The medium was refreshed every 3 days till the second week when the suspended cells were removed and the attached cells were allowed to expand to reach the confluence of 80%. Isolated cells were characterized by flow cytometry (BIO-RAD, USA) considering CD38 and CD105 as positive mesenchymal stemness markers and CD34 as a negative marker.

To collect conditioned medium from the stem cells, 10 ml serum-free DMEM was added to each flask during passages 3–7 and incubated for 48 h. Afterward, the culture medium was collected and centrifuged at 500 rpm for five min to remove cellular debris. Finally, the relevant medium was concentrated 20 times by the lyophilizer and kept at a temperature of − 80 °C until use.

### 2.4 PRP preparation

Blood (5 ml) was collected from the left ventricle of ten selected rats. Sodium citrate (3.8%) was used at a ratio of 9:1 to prevent blood clotting. Two steps of centrifugation were then performed. The first stage involved centrifugation at a speed of 1000 rpm for 15 min. After this stage, the tubes had three distinct parts: the upper, middle, and lower parts corresponding to plasma, buffy coat, and red blood cells, respectively. The plasma and buffy coat were transferred to another tube followed by centrifugation at 12,000 rpm for 5 min. After this step, the upper two-thirds part which is platelet-poor plasma (PPP) was discarded while the lower third contains PRP solution. The PRP was left at room temperature for one hour. It was then stirred for one hour at 60 rpm to separate the platelets and evenly disperse them in the liquid. They were placed in a freezer until use.

### 2.5 Behavioral tests

#### 2.5.1 Morris water maze (MWM)

The MWM test is used to examine spatial memory, which is a circular tank with a black wall filled with water. It consists of a pond with approximate width and depth of 120 and 50 cm, respectively, and filled with water to a height of 30 cm. The water temperature is kept at 25 °C. A dark platform was used as a hidden platform, placed in the middle of one of the four quadrants of north, south, east, and west, at a distance of 1.5 cm beneath the water level. This platform is just a place for the animal to escape from the water. The walls around the maze need to have objects and signs with different geometric shapes to help the rat in catching the platform.

The movement and behavior of the animal are monitored in a dark room by a special camera located vertically above the central area of the tank. The information is obtained by Ethovision (Noldus Information Technology, Netherlands) software specifically designed for recording. Before starting the experiment, the animals swam for 60 s to adapt to platform-free conditions. The duration of this experiment was 4 days, the first three days were considered as a training test while the last day was taken as the probe test. During the training tests, the platform was fixed for three days (each day consisted of four trials). In each trial, the animal was dropped from one of the quarters with its face toward the wall of the pool. The animal was randomly released as determined by the software. The animal was permitted to swim for 60 s to catch the hidden platform. If it failed, the researcher guided it to catch the platform. The animal was allowed to sit on the platform for 30 s and then, it was again dropped from another quadrant. The time taken to reach the platform, the traveled distance during this time, and the swimming speed of the animal were calculated by the software. After 4 trials, the animals were returned to their cages with dry towels. During the probe test, the platform was removed and the rats dropped from the other side of the platform quadrant to swim for 60 s to analyze how long it takes for the rats to cross the platform site (Aghaei et al., 2023).

#### 2.5.2 Novel objective recognition (NOR)

The NOR test is a recognition memory test that is performed in two days in a white box. On the first day, the rats are placed in a box with no external objects to get acquainted with the environment. On the second day or the day of the experiment, two identical objects are placed near the two corners of the box at the same distance from it’s the wall. Rats are free to explore the objects and all parts of the box for 10 min. After an hour, the rats are returned to the box where a new object is replaced with one of the previous objects. Then, the time passed around the new and old objects in 5 min is measured to determine the discrimination ratio (the time passed around the new object minus the old one divided by the time spent exploring around the two objects) and total exploration time (Zarifkar et al., 2019).

### 2.6 Tissue preparation

The rat brain fixation was performed by the perfusion method (Sotoudeh et al., 2020). In short, the animals were anesthetized by ketamine and xylazine. Then, the perfusion was done by injection of 0.1 M PBS and 4% paraformaldehyde through the left ventricle (i.e. transcardial perfusion). Thereafter, the brain was removed and immersed in the 4% paraformaldehyde for 24 h at 4°C. The samples were then transferred to 30% sucrose solution for 72 h at 4°C and retained at − 80°C. After that, the brain tissue was maintained at an ideal cutting temperature and cut into a 50 µm coronal section thickness by cryostat (Leica CM1860, Germany). Every two sections were immersed in glycerin solution.

The sections were stained with cresyl violet for stereological study. In short, the slide tissue sections were preceded with xylene, hydrated in descending series of ethanol (100–70%), rinsed with distilled water, and stained with 0.1% cresyl violet acetate for 5 min. Then the sections were subsequently dehydrated in a rising set of ethanol (70–100%), cleared with xylene, and mounted.

### 2.7 Estimation of volume

**Fig. 6:**
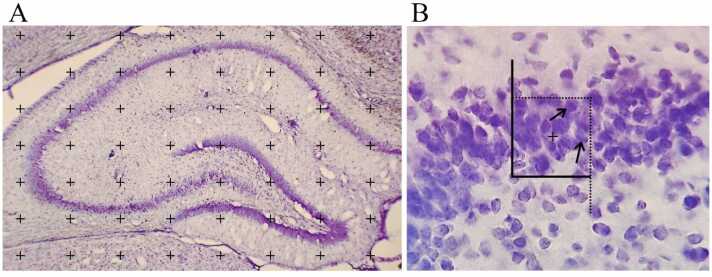
A. Cavalieri method is used to measure the CA1 and DG volume B. Disector method is used to count the pyramidal and granular cells in the hippocampus. The black arrows show the neurons which they are counted in frames.

The volume (V) was measured by using an optical microscope and stereological software (StereoLite, SUMS, Shiraz, Iran) developed at the Histomorphometry and Stereology Research Center according to the Cavalieri method. For determining the total volume of the CA1 and DG of the hippocampus, 8–10 sections were considered. Using the grid points and the points were counted within the region of interest (Fig. 6A). Then, volume was calculated by the following formula:

V= ∑p·a/p·t

Where, “∑P” is the total points on the area of samples, “a/p” represents the area of each point, and “t” denotes the interval between the sections.

### 2.8 Estimation of the cell number

The identical sections considered for measuring the volumes were used to calculate the number (N) of pyramidal and granular cells based on the optical disector method using an optical microscope (E-200, Nikon, Japan) with the help of oil immersion objective lens (40x, numerical aperture=1.3) and stereological software (StereoLite, SUMS, Iran). An impartial counting frame with inclusion and exclusion margins was overlaid on the pictures. The nuclei of cells placed entirely or partially within the inclusive area of the counting frame were counted (Fig. 6 B). The z-axis position change (depth) was evaluated by a microcator (MT12, Heidneheim, Germany) attached to the microscope stage. The first 5 µm distance was ignored as a guard zone to achieve unbiased counting results.

$$N=\sum Q/\sum P\times (\frac{a}{f})\times h$$

In the above equation, ΣQ” shows the number of the counted nuclei, “ΣP” represents the number of counting frames in all fields, a/f stands for the area per frame, and “h” denotes the disector height (Sotoudeh et al., 2020, Asadi-Golshan et al., 2021).

### 2.9 Statistical analysis

Data are expressed as mean ± SEM and the significant level is P < 0.05. Quantitative data are analyzed by GraphPad Prism (version 7.1, USA) using nonparametric tests, including Kruskal Wallis and Mann-Whitney test. One-way ANOVA test followed by Tukey’s multiple comparison test is employed for all data except for the escape latency and swimming distance in the MWM which is used two-way ANOVA.

## 3 Results

### 3.1 Body weight

The body weight of the rats in the experimental groups was shown in Fig. 1B. At the beginning of the experiment, there was no significant difference between groups while after AD induction, significant weight loss was observed in the AD group, and there was a significant difference between AD and sham groups (p < 0.001). On the other hand, treatment with CM or CM+PRP prevented weight loss in these groups compared to the AD group (p < 0.01).

### 3.2 Recognition memory test

**Fig. 2:**
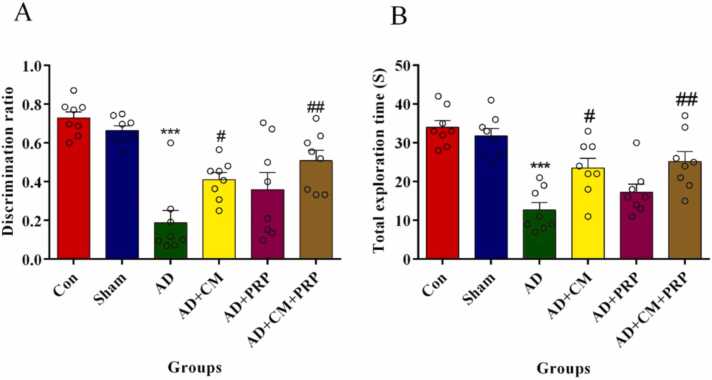
NOR test results are shown as mean ± SEM (n = 8). (A) Discrimination ratio and (B) Total exploration time during the testing phase. Data are analyzed by one-way ANOVA followed by Tukey’s multiple comparison test. * ** Significant difference with the Sham group (p < 0.0001). # Significant difference with AD group (p < 0.01). ## Significant difference with AD group (p < 0.001). Con: Control, AD: Alzheimer’s Disease, CM: Conditioned Medium, PRP: Platelet-Rich Plasma.

Fig. 2A and B show the difference in the NOR results of sham, AD, and treatment groups. The discrimination ratio and total exploration time in the AD and PRP groups are significantly decreased compared to the other groups. In both groups, rats spent less time than other groups around the novel object. However, the application of CM and CM+PRP as a treatment reversed the STZ-induced impairment and the rats spent longer around the new object (p < 0.01, Fig. 2). It means the simultaneous use of CM and PRP could decrease the side effect of AD.

### 3.3 Spatial memory test

**Fig. 3:**
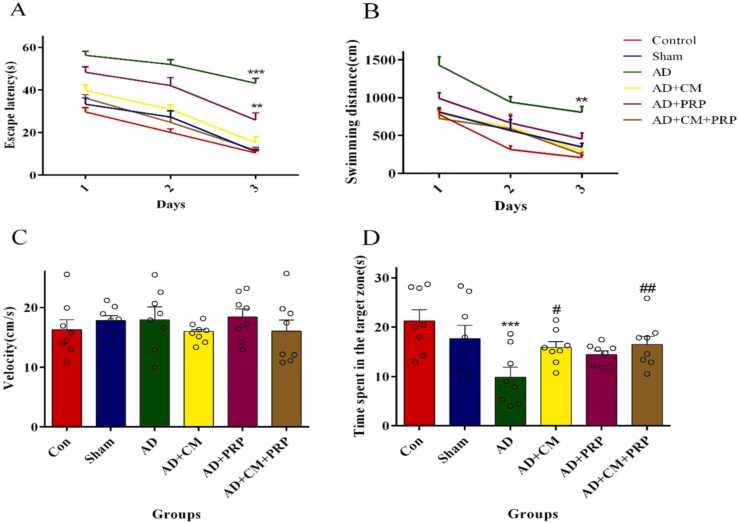
MWM test results are shown as mean ± SEM (n = 8). (A) Escape latency, (B) swimming distance (C) Mean swimming velocity, and (D) Time spent in the target zone. Data were analyzed by two-way ANOVA followed by Tukey’s multiple comparison tests for graphs A and B while one-way ANOVA was used for graphs C and D.*** and ** Significant difference with the Sham group (p < 0.0001 and p < 0.001 respectively). # Significant difference with the AD group (p < 0.01). ## Significant difference with AD group (p < 0.001). Con: Control, AD: Alzheimer’s Disease, CM: Conditioned Medium, PRP: Platelet-Rich Plasma.

The difference in the spatial memory and learning results of the sham, AD, and treatment groups was assessed by the MWM test as illustrated in Fig. 3. Fig. 3A and B represent the learning ability of animals during three consecutive days of training to find the hidden platform. These figures demonstrate a negative linear relationship between the training days and escape latency across the groups.

Fig. 3A shows the escape latency to reach the hidden platform. Two-way ANOVA was used to compare the behavior of the rats on different trial days. The results indicated a significant difference between the AD and PRP groups with other groups. The AD and PRP groups exhibited significantly lower spatial learning ability from the first to the third day. Therefore, the escape latency did not decrease in the AD and PRP groups as much as in the other groups.

Fig. 3B shows the swimming distance of different groups to catch the platform. The path distance decreased by learning about the location of the platform during the trial days. The decline in the swimming distance of the AD and PRP groups was not as much as other groups during the three consecutive trial days. This trend implies that the rats in the AD and PRP groups cannot find the hidden platform within a short distance, therefore, they should swim a longer path to reach the platform. A significant difference was also observed between the CM+PRP and AD group whose data are almost near the Sham group data.

Fig. 3C shows the mean velocity in all the groups during the training days. One-way ANOVA results do not exhibit any significant differences among groups, which means that the swimming speed did not affect the performance of the animals.

Fig. 3D demonstrates a remarkable decline in the time spent in the target zone and its proximity for the AD group, while the CM treatment with and without PRP reversed the memory impairment. The single-use of PRP caused no significant difference compared to the AD group.

### 3.4 Stereological results

#### 3.4.1 Volume of CA1 and DG

**Fig. 4:**
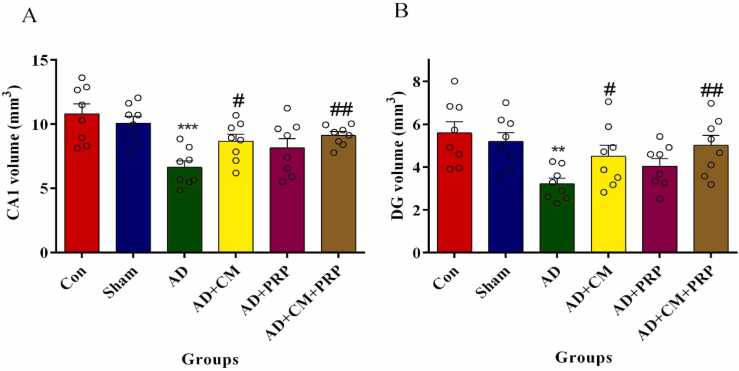
The total volume of CA1 (A) and DG (B). Results are shown as mean ± SEM (n = 8). Data are analyzed by one-way ANOVA followed by Tukey’s multiple comparison test. *** and ** Significant difference with the Sham group (p < 0.0001 and p < 0.001 respectively). # Significant difference with the AD group (p < 0.01). ## Significant difference with the AD group (p < 0.001). Con: Control, AD: Alzheimer’s Disease, CM: Conditioned Medium, PRP: Platelet-Rich Plasma.

As Fig. 4 A and B show, the total volume of CA1 and DG was significantly reduced in the AD group compared to the sham group (p < 0.0001). But no significant difference was observed between AD and PRP groups, while injection of CM and CM+PRP could significantly raise the volume of CA1 and DG.

#### 3.4.2 Number of pyramidal and granular cells of the hippocampus

**Fig. 5:**
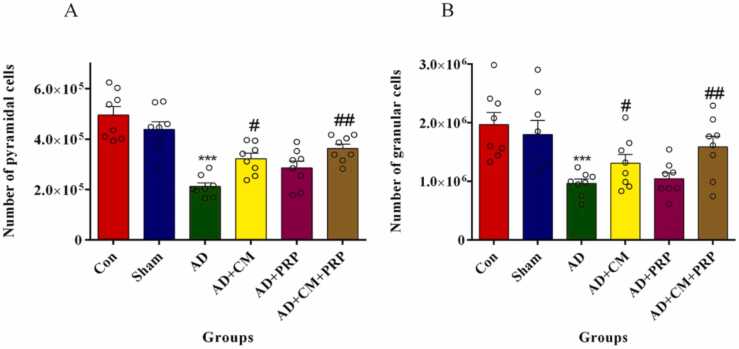
The total number of pyramidal cells in CA1 (A) and granular cells in DG (B). Results are shown as mean ± SEM (n = 8). Data are analyzed by one-way ANOVA followed by Tukey’s multiple comparison test. *** Significant difference with the Sham group (p < 0.0001). # Significant difference with the AD group (p < 0.01). ## Significant difference of the AD group (p < 0.001). Con: Control, AD: Alzheimer’s Disease, CM: Conditioned Medium, PRP: Platelet-Rich Plasma.

**Fig. 7:**
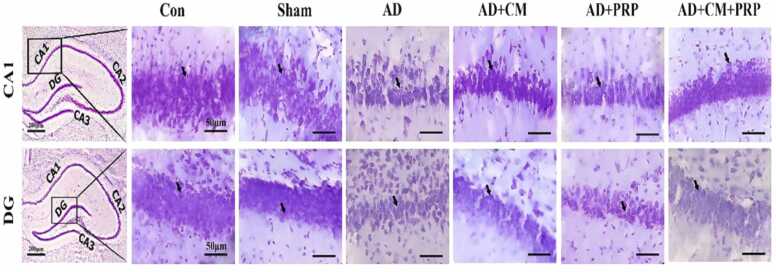
Representative photographs of the cresyl violet stained section show the number of pyramidal and granular cells. It showed that the neurons in both areas decreased in the AD group compared to the sham group while in the CM and CM+PRP groups, the number of neurons increased significantly. The black arrows show the pyramidal and granular cells in CA1 and DG respectively. Con: Control, AD: Alzheimer’s Disease, CM: Conditioned Medium, PRP: Platelet-Rich Plasma.

The total numbers of pyramidal cells of the CA1 (Fig. 5A) and granular cells of the DG (Fig. 5B) were considerably reduced in the AD group compared to the sham group. The results also show that the co-injection of CM and PRP can improve the side effect of AD by significant enhancement of the total number of pyramidal and granular cells (Fig. 7).

## 4 Discussion

The findings of this study revealed that STZ could induce AD in rat models by decreasing their memory and learning and causing structural changes. In the AD group, the discrimination ratio and total exploration time decreased in the NOR test while the escape latency and swimming distance showed no decrease compared to the Sham group. In line with our findings, previous studies showed that the injection of STZ into the lateral ventricle of the rat brain could result in memory damage and create a histopathological effect similar to sporadic AD (Chen, 2016, Choi et al., 2014). STZ could also decrease the glucose/energy metabolism in the brain of rodents and produce oxidative stress with adverse effects on neurons, maintenance of synaptic activity, and release of neurotransmitters in hippocampal neurons (Arluison et al., 2004, Young and McKenzie, 2004). Therefore, AD rats could not learn how to find the platform in MWM and remember old and new objects in the NOR tests.

A decrease in learning and memory (indicated by the behavioral study) in AD rats affected the hippocampal structure. In agreement with this research, studies have reported hippocampal atrophy and a reduction in the volume of the hippocampus in AD (Risacher et al., 2009, Uysal and Ozturk, 2020). The stereological findings revealed ∼41% and 38% reduction in the volume of CA1 and DG in the AD group compared to the Sham group, respectively. Similar to the West’s findings which introduced CA1 as the most vulnerable region of atrophy in AD (West et al., 1994); in this work, CA1 was more vulnerable than DG. These data confirmed the findings of Ugolini et al. who mentioned that the lesion of CA1 is enough for memory impairment (Ugolini et al., 2018). Moreover, memory showed a linear relationship with the number of neurons in the hippocampus. Edler et al. (2020) reported neuronal loss in chimpanzees as they get older. They also reported a 12% decline in the neuronal density of CA1 by aging (Edler et al., 2020). The pyramidal cells of the CA1 and granular cells of the DG play a key role in learning and memory (Lopez-Rojas and Kreutz, 2016, Yáñez Santamaría, 2018). Our data indicated a 51% and 46% decline in the number of pyramidal and granular cells of the AD group as compared to the sham group. These results agreed with another study showing a decline in the total number of neurons in AD compared to healthy groups (Pelvig et al., 2003). The hippocampus volume will be decreased in AD due to a decrement in the number of neurons, thus, the patients cannot learn and remember like the healthy people.

Researchers are looking for the best treatment for AD and its related problems. Although some studies showed that PRP has a neuroprotective effect by producing growth factors, cytokines, and chemokines (Hu et al., 2022), others disagree with these findings. They mentioned the unprotective effect of platelets is due to the secretion of ß amyloid peptide following platelet activation (Leiter and Walker, 2020). Moreover, ß amyloid is involved in platelet activation and induces Reactive Oxygen Species (ROS) production which is not helpful to treat AD (Abubaker et al., 2019, Gowert et al., 2014). The findings of the present study showed that the sole use of PRP cannot improve hippocampus damage based on the behavioral tests and stereological analysis. Demir et al. mentioned that intravenous injection of PRP could improve learning and memory in elderly mice (Demir and Karagoz, 2020). It seems that PRP might be beneficial for elderly animals but it does not have any positive effect on AD. Bayat et al. (2020) indicated that the late injection of PRP cannot improve memory and does not have any beneficial effect on long-term potential. They mentioned that the number of injections might be vital (Bayat et al., 2020). Karina et al. showed no side effects from intravenous administration of PRP in patients with pathological problems (Karina et al., 2021). In line with this research, some studies mentioned the beneficial effects of PRP on AD; while others reported negative effects of PRP which contradict our results. Hence, further investigations are required for the molecular investigation of PRP through vein.

Some scientists have noticed the beneficial effect of CM in memory loss improvement, which agrees with our findings. In this study, CM could cease the atrophy of CA1 and DG by increasing the number of neurons, manifested as an improvement in behavioral tests. Zhang et al. (2017) showed that CM can support retinal neurons and photoreceptor differentiation (Zhang et al., 2017). Xiao et al. (2021) used extracellular vesicles from human umbilical cord mesenchymal stem cells to treat spinal cord injury (SCI) in rats. They mentioned that CM could reduce pathological changes, improve motor function, and promote nerve function repair (Xiao et al., 2021) which agreed with our results on the ability of CM to improve memory by increasing the number of neurons in AD. Such beneficial effects on memory could be assigned to numerous growth factors which can help neurons repair. The insulin receptor (IR) is one of the major receptors in the neurons with a decisive role in energy homeostasis and glucose maintenance (Talbot, 2014). STZ causes insulin resistance by reducing IR signaling through autophosphorylation which promotes AD (Sun et al., 2018). On the other hand, IGF is decreased in AD, promoting the progress of AD (Westwood et al., 2014, Galle et al., 2020). Therefore, the IGF content of CM could help neurons to restore and increase the volume of the hippocampus and accelerate behavioral improvement in AD.

Our results showed that injection of combined CM and PRP could increase memory improvement, volume, and the number of neurons in CA1 and DG compare to the AD group. Whereas it wasn’t significant difference between CM and CM+PRP groups. Consequently, based on our findings CM is as effective as stem cell therapy in the treatment of AD. Further studies require a deeper understanding of the cooperative mechanisms of CM and PRP in AD.

## 5 Conclusion

Behavioral tests and stereological structures of the hippocampus in the present study indicated a reduction in the volume and the quantity of neurons in the AD group. The behavioral tests showed an increment in the time required for the animal to find the platform. The administration of CM with or without PRP led to protective effects in the AD model rats. Intravenous injection of PRP alone did not improve the side effects of AD. However, further studies are required to find the best treatment to improve memory.

## Ethical statement

Informed consent was attained from all full-term women donors, who underwent an elective cesarean for collecting umbilical cord. The project was approved based on the guidelines of the Animal Care and Ethics Committee of the Shiraz University of Medical Sciences Shiraz, Iran (approval No. IR.SUMS.REC.1400.047).

## CRediT authorship contribution statement

Conception and design: Amin Firoozi, Farzaneh Dehghani. Data acquisition: Amin Firoozi, Aliakbar Alizadeh, Asadollah Zarifkar, Mohammad Reza. Namavar, Omid Alavi. Data analysis and interpretation of data: Amin Firoozi, Aliakbar Alizadeh. Drafting of the manuscript: Amin Firoozi. Critical revisions: Amin Firoozi, Farzaneh Dehghani, Tahereh Esmaeilpour, Mohammad. Reza Namavar. Final approval: all authors.

## Declaration of Competing Interest

The authors declare no competing financial interests.

## Data Availability

The experimental data used to support the findings of this study are available from the corresponding author upon request.
